# Comparison of vascular risk profile and clinical outcomes among patients with central (branch) retinal artery occlusion versus amaurosis fugax

**DOI:** 10.1186/s42466-024-00326-3

**Published:** 2024-05-16

**Authors:** Norma J. Diel, Stefan T. Gerner, Thorsten R. Doeppner, Martin Juenemann, Toska Maxhuni, Tobias Frühwald, Andre Worm, Omar Alhaj Omar, Lyubomyr Lytvynchuk, Tobias Struffert, Pascal Bauer, Hagen B. Huttner

**Affiliations:** 1 Department of Neurology, University Hospital Giessen, Klinikstrasse 33, 35392 Giessen, Germany; 2 Center for Mind, Brain and Behavior (CMBB), University of Marburg, Marburg, Germany; 3 Department of Neuroradiology, University Hospital Giessen, Giessen, Germany; 4 Department of Ophthalmology, University Hospital Giessen, Giessen, Germany; 5 Department of Internal Medicine I, Division of Cardiology and Angiology, University Hospital Giessen, Giessen, Germany

**Keywords:** Central retinal artery occlusion, Amaurosis fugax, Ischemic stroke, Transient ischemic attack, Intravenous thrombolysis

## Abstract

**Background:**

Retinal artery occlusions lead to sudden, painless vision loss, affecting millions globally. Despite their significance, treatment strategies remain unestablished, contrasting with acute ischemic stroke (AIS), where IVT has proven efficacy. Similar to AIS, retinal artery occlusions demand urgent evaluation and treatment, reflecting the principle "time is retina". Even for patients with transient monocular vision loss, also known as amaurosis fugax (AF), pertinent guidelines meanwhile recommend immediate emergency assessment in a specialized facility. However, data on the clinical benefit and comparability with persistent occlusions are missing. This study aimed to compare the results of a comprehensive stroke-workup among patients with persistent retinal artery occlusions (RAO), including both central retinal (CRAO) and branch retinal artery occlusion (BRAO) and those with AF.

**Methods:**

Conducted at the University Hospital Giessen, Germany, this exploratory cross-sectional study enrolled patients with transient or permanent unilateral vision loss of non-arteritic origin. The primary outcome were differences between the two groups RAO and AF with regard to cardiovascular risk profiles and comorbidities, vascular and pharmacological interventions and clinical neurological and ophthalmological outcomes. Secondary outcome was a sub-group analysis of patients receiving IVT.

**Results:**

Out of 166 patients assessed, 76 with RAO and 40 with AF met the inclusion criteria. Both groups exhibited comparable age, gender distribution, and cardiovascular risk profiles. Notably, RAO patients did not show significantly more severe vascular comorbidities than AF patients. However, AF patients received vascular interventions more frequently. Pharmacological intervention rates were similar across groups. RAO patients had slightly worse neurological outcomes, and IVT did not yield favorable ophthalmological outcomes within any observed patients.

**Conclusion:**

The study found similar vascular burden and risk factors in patients with RAO and AF, with implications for clinical workflows. IVT for RAO may only be effective in very early treatment windows. This emphasizes the need for public awareness and collaborative protocols between ophthalmologists and neurologists to improve outcomes.

## Background

Retinal artery occlusions are diseases affecting the central retinal artery (CRAO), or branches respectively (i.e. BRAO), and lead to a painless, sudden, usually unilateral irreversible severe vision loss or, in the case of BRAO, partial loss of visual field [17]. Incidence of RAO is age-dependent ranging between 2.7–4.5/100,000 people with a steep increase up to 57/100,000 in patients above 80 years [34]. Main causes of CRAO and BRAO, in the following summarized as retinal artery occlusions (RAO), include thrombo-embolism caused by carotid plaques or atrial fibrillation. The EAGLE study revealed elevated cardiovascular risk factors including obesity, hypertension, smoking, hypercholesterolemia, and diabetes as well as cardiac arrhythmia in up to 20% of patients with RAO [16, 38]. Contrary to acute ischemic stroke (AIS) in which intravenous thrombolysis (IVT) is considered to be a first line therapeutic approach, there are up to now no established proven treatment strategies for RAO [32]. In spite of local and systemic treatment modalities, the prognosis regarding recovery of vision is quite unfavorable with a majority of patients improving only little or not at all [17]. However, the new surgical and laser assisted approaches show promising anatomic and functional results when applied on time [27]. Nevertheless, there is a call for action to prioritize these patients like strokes as neurological emergencies [15, 36].

These permanent monocular vision losses are distinguished from those with transient monocular vision losses of vascular origin, i.e. amaurosis fugax (AF). AF may occur both because of thromboembolism or due to retinal hypoperfusion on the basis of proximal extracranial perfusion deficits, and is associated with increased risk for subsequent stroke [12]. In simple terms, RAO can be considered the ophthalmological equivalent of AIS, while AF represents the ophthalmological equivalent of a transient ischemic attack (TIA), as stated by the American Heart Association [14].

In both diseases, the likelihood of concomitant and subsequent cerebral ischemia is increased [33], why available guidelines suggest to consider both, AF and RAO, as neurological emergencies, i.e. “time is retina”, with immediate presentation to an (neurological) emergency department (ED) to timely realize neuroimaging and eventually IVT [9, 13, 36, 39]. The presentation of RAO- and AF-patients is often delayed due to an initial presentation to the ophthalmologist, which undermines these recommendations in clinical reality [39].

For AIS and TIA, there is some limited data that suggests that patients may differ in their risk profiles, and that patients with TIA tend to be younger and to suffer from a lower prevalence of vascular risk factors such as peripheral arteriopathy [22, 42]. In contrast, only few data exist regarding the vascular risk profile among patients with RAO versus AF. Thus, the aim of this study was to compare cardiovascular risk profiles and comorbidities among patients with RAO and AF, and thus to substantiate the demand on an immediate presentation in the ED. Further, we a priori decided, to sub-analyze time windows and clinical outcomes among the subgroup of patients with RAO receiving IVT.

## Methods

### Study design, study population and clinical parameters

We performed an exploratory cross-sectional study at the tertiary care center of the University Hospital Giessen, Germany. The study protocol was approved by the local institutional review board (Giessen Stroke Registry: GIST. ClinicalTrials.gov Identifier: NCT05295862). GIST is a registry in which all patients with cerebral strokes but also patients with retinal ischemia treated at the University Hospital Giessen are consecutively included. Over a two-year period, data on all consecutive patients with symptoms of transient or permanent unilateral vision loss of non-arteritic origin were entered into an observational database. Patient characteristics, CRAO-/BRAO- and AF-specific information, and medications were recorded. Patients whose visual loss was caused by an anterior or posterior ischemic optic neuropathy (AION/PION) were excluded. We enrolled all patients with persistent (CRAO/BRAO) or transient (AF) vision loss for analysis. The variables collected for the two patient groups included socio-demographic characteristics, parameters on cardiovascular risk profile and comorbidities, according to established criteria [2, 6, 11, 30, 37, 44], clinical and radiological data and results of ophthalmological and neurological examinations. While the data collection itself was thus prospective, the development of the analyses described here, i.e. the selection of variables and the compilation of an evaluation plan, only took place after the data collection and was therefore retrospective.

### Ophthalmological and neurological outcomes

For grading of vision loss we used International Classification of Diseases 11th edition criteria, as suggested by WHO. Neurological impairment was obtained according to the National Institutes of Health Stroke Scale (NIHSS), and neurological outcome was assessed using the modified Rankin Scale (mRS) at hospital discharge. Furthermore, data on imaging and neurovascular diagnostics as well as treatment data, i.e. surgery or invasive interventions, and mode and intensity of pharmacological secondary prophylaxis were assessed.

### Ultrasound workup

Three types of ultrasound examinations were performed on the study population including carotid ultrasound and transthoracic/transesophageal echocardiography (TTE/TEE) in all patients, as well as transocular ultrasound in RAO patients. Outcomes were: Evidence of atherosclerosis, ipsilateral internal carotid artery (ICA) stenosis ≥ 50%, spot sign, patent foramen ovale (PFO) and the ejection fraction (EF) [3, 19], North American Symptomatic Carotid Endarterectomy [31].

### Endpoints

Endpoints consisted of: Analysis of (i) vascular interventions (defined as either ICA stenting or carotid endarterectomy (CEA)), (ii) pharmacological interventions (defined as either no change on antithrombotic medication vs. intensification, the latter reflecting initiation of antithrombotic treatment in naive patients, dual antiplatelet therapy (DAPT) in previously acetylsalicylic acid (ASA) monotherapy patients or initiation of oral anticoagulation (OAC; direct oral anticoagulants or warfarin)), as well as (iii) neurological clinical outcome at discharge (NIHSS and mRS). We assessed (iv) ophthalmological outcome after IVT in the sub-group analysis of patients with CRAO/BRAO.

### Statistical analysis

Descriptive statistics were employed to summarize data on clinical characteristics, stroke work-up and procedural outcomes of the two patient populations. These statistics included means, standard deviations (SD) and range for continuous variables, and frequencies and percentages for categorical variables. To assess the normality of the distribution of continuous variables, the Shapiro–Wilk test was applied. Normally distributed data were presented by mean (SD) and compared using two-sided t-tests, non-normally distributed data were presented by median (range) and compared using the Mann–Whitney U-test and Wilcoxon signed-rank test, respectively. The significance level was set at alpha = 0.05 with a statistical trending in cases of < 0.1. Categorical variables were analyzed using the Chi-squared test, and Fisher's Exact Test, respectively. All statistical analyses were conducted using SPSS Version 29.0 (www.spss.com).

## Results

### Participants

**Fig. 1:**
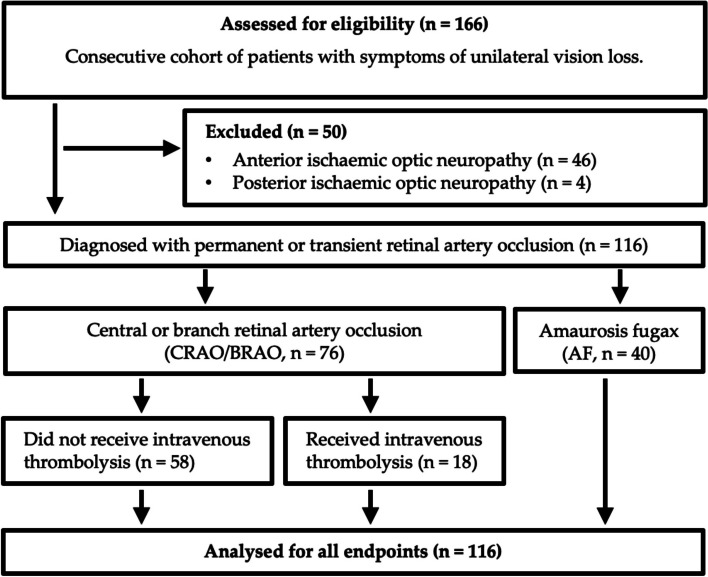
STROBE flow diagram of study participants

A total of 166 patients presented to the ED with symptoms of unilateral vision loss during the defined study period and were therefore assessed for eligibility. Patients whose symptoms were attributable to AION (*n* = 46) or PION (*n* = 4) were excluded. Of the remaining patients, 76 patients met the diagnostic criteria for permanent (CRAO/BRAO) and 40 patients for transient (AF) retinal arterial occlusion (Fig. 1).

### Baseline clinical data

Table 1 shows the baseline sociodemographic and clinical characteristics. The RAO and AF groups had a comparable distribution of age, gender and affected sides. Based on the TOAST criteria [1], there was no significant difference in the distribution of etiology among both groups. In general, patients with RAO showed a tendency towards a higher cardiovascular risk profile and correspondingly a higher use of respective premedication. The proportion of patients with antithrombotic therapy or intake of OAC was not significantly different among both groups, as were the majority of laboratory parameters on admission (Table 2).

**Table 1:** Demographics and baseline clinical data

Characteristics	RAO (*n* = 76) (CRAO ( *n* = 47)/BRAO ( *n* = 29))	AF (*n* = 40)	*P*-value
Female^a^	32 (42.1)	13 (32.5)	0.313
Age (years)^b^	71.9 (11.3/35–89)	70.9 (14.1/36–93)	0.862
Right sided^a^	44 (57.9)	27 (67.5)	0.313
TOAST^a^			0.603
* Large artery atherosclerosis*	22 (28.9)	14 (35)	
* Cardioembolism*	7 (9.2)	2 (5)	
* Small vessel disease*	28 (36.8)	12 (30)	
* Other determined cause*	5 (6.6)	1 (2.5)	
* Undetermined cause*	14 (18.4)	11 (27.5)	
Pre-mRS (0–5)^c^	0 (0–0)	0 (0–0)	0.451
**NIHSS on admission (0–42)**^**c**^	**1 (0–1)**	**0 (0–0)**	** < 0.001**
**Visual impairment (VI)**^**c**^	**3 (1–4)**	**0 (0–0)**	** < 0.001**

*RAO* Retinal artery occlusion, *CRAO* Central retinal artery occlusion, *BRAO* Branch retinal artery occlusion, *AF* Amaurosis fugax, *TOAST* Trial of Org 10,172 in Acute Stroke Treatment, *NIHSS* National Institutes of Health Stroke Scale, *mRS* Modified Rankin scale, *SD* Standard deviation, *IQR* Interquartile range

^a^*n* (%)

^b^mean (SD/range)

^c^median (IQR)

**Table 2:** Comorbidities and baseline laboratory results

Characteristics	RAO (*n* = 76) (CRAO ( *n* = 47)/BRAO ( *n* = 29))	AF (*n* = 40)	*P*-value
Comorbidities			
BMI (kg/m^2^)^b^	27.8 (4.6/17.3–41.4)	26.8 (4.04/19.1–35.4)	0.358
Arterial hypertension^a^	63 (82.9)	27 (67.5)	0.059
Diabetes mellitus^a^	23 (30.3)	6 (15)	0.071
Hypercholesterolaemia^a^	49 (64.4)	26 (65)	0.971
Previous stroke^a^	12 (15.8)	5 (12.5)	0.634
Coronary artery disease^a^	17 (22.4)	7 (17.5)	0.538
Congestive heart failure^a^	10 (13.3)	2 (5)	0.212
Peripheral arterial occlusive disease^a^	10 (13.2)	4 (10)	0.768
Chronic kidney disease^a^	18 (23.7)	8 (20)	0.651
Atrial fibrillation^a^	16 (21.1)	3 (7.5)	0.061
Prior medication^a^			
Antiplatelet agents			0.25
*ASA*	30 (39.5)	10 (25)	
*DAPT*	1 (1.3)	1 (2.5)	
Oral anticoagulation	14 (18.4)	5 (12.5)	0.413
Statin	33 (43.4)	14 (35)	0.38
Baseline laboratory results^b^			
Hemoglobin (g/L)	139 (17.6/80–186)	139.6 (15.6/91–164)	0.891
Leucocytes (10^9/L)	8.8 (2.6/4.7–18.1)	7.9 (1.8/4.1–11.6)	0.081
Thrombocytes (10^9/L)	257 (84/123–574)	233.2 (72.7/139–558)	0.071
International Normalized Ratio	1 (0.4/0.7–3.2)	0.9 (0.1/0.8–1.1)	0.108
Serum Creatinine (mg/dL)	1.1 (0.8/0.5–5.6)	1.1 (1.3/0.5–8.7)	0.923
**C-reactive protein (mg/L)**	**12.6 (27/0–166.3)**	**3.3 (4.3/0–18.1)**	**0.024**
Blood Glucose (mg/dL)	121.8 (30.6/75–229)	123 (27.1/82–198)	0.616
GFR (ml/min/1.73m^2^)	79.1 (30.8/10.3–136.1)	82.9 (28.2/5.1–133.3)	0.609
HbA1c (%)	6.1 (0.7/5.1–8.1)	5.9 (0.6/5–8.5)	0.09
Triglycerides (mg/dL)	144.4 (86.7/38–583)	121.1 (64.7/46–359)	0.106
Total cholesterol (mg/dL)	177 (41.3/104–281)	182.9 (41/108–293)	0.482
HDL-C (mg/dL)	55.6 (28.8/23–159)	50.1 (19.5/26–126)	0.564
LDL-C (mg/dL)	106.8 (45.5/21–206)	121.4 (44.3/47–238)	0.112

*RAO* Retinal artery occlusion, *CRAO* Central retinal artery occlusion, *BRAO* Branch retinal artery occlusion, *AF* Amaurosis fugax, *ASA* Acetylsalicylic acid, *DAPT* Dual antiplatelet therapy, *BMI* Body mass index, *GFR* Glomerular filtration rate, *HDL-C* High-density lipoprotein-cholesterol, *LDL-C* Low-density lipoprotein-cholesterol, *SD* Standard deviation

^a^*n* (%)

^b^mean (SD/range)

Key findings in disadvantage of RAO, as compared to AF, were a significantly elevated baseline C-reactive protein (CRP) value (12.6 vs 3.3 mg/L) and, as per definition, ophthalmological and neurological deficits an admission. Both groups had similar pre-existing mRS-scores. The majority of patients with RAO (57 (75%)) presented later than 4.5 h after symptom onset. Among the patients with AF, about half (17 (42.5%)) had a symptom duration of less than 5 min. Within the RAO group, 46 (62%) patients met the WHO criteria for blindness in the affected eye [7].

### Vascular diagnostics

Both patient groups received a comparable workup and thus the same vascular diagnostics. Transocular ultrasound was performed in 51 (67%) patients of the RAO group with a spot sign evident in 15 (29.4%) patients. Regarding carotid ultrasound we observed a trend towards higher amount of atherosclerosis in RAO patients (95.8% vs. 83.8%), while AF-patients showed an ipsilateral ICA stenosis ≥ 50% (26.3% vs. 12.3%) more frequently. Cardiac workup revealed no differences between RAO versus AF with respect to mode of sonography (TTE/TEE), incidence of PFO (14% vs. 40%), and EF (mean: 57 vs. 56%).

### Rates of vascular intervention, antithrombotic medication and neurological outcome

Table 3 summarizes the results of the interventions performed, and secondary prophylactic measures initiated, during the period of hospitalization. Compared to RAO, patients with AF received significantly more vascular interventions (4 (5.3%) vs. 7 (17.5%); *p* < 0.05). Regarding pharmacological interventions, intensification in medication was not significantly different among both groups (46/76 vs. 29/40). There was no signal among the sub-group analysis of patients with platelet inhibitors *versus* OAC for imbalances in favor or against of any of the studied groups. There was a significant difference in the median mRS Score to the disadvantage of patients with RAO (1 (0–1) vs. 0 (0–0); Table 3).

**Table 3:** Treatment and outcome

Characteristics	RAO (*n* = 76) (CRAO ( *n* = 47)/BRAO ( *n* = 29))	AF (*n* = 40)	*P*-value
Intravenous thrombolysis^a^	18 (23.7)		N/A
* Door-to-needle-time (minutes)*^b^	52.2 (47.2/20–198)		N/A
**Vascular intervention**^**a**^			**0.05**
* Internal carotid artery—Stenting*	0	1 (2.5)	
* Carotid endarterectomy*	4 (5.3)	6 (15)	
Medication at discharge^a^			
Antiplatelet agents			0.314
*ASA*	53 (69.7)	27(67.5)	
*DAPT*	10 (13.2)	9 (22.5)	
*Change in antiplatelet therapy*	41 (53.9)	29 (72.5)	0.052
Oral anticoagulation	19 (25)	5 (12.5)	0.114
*New on oral anticoagulation*	5 (6.6)	0	0.163
Statin	71 (93.4)	40 (100)	0.163
*New on Statin*	38 (50)	26 (65)	0.123
**NIHSS at discharge (0–42)**^**c**^	**1 (0–1)**	**0 (0–0)**	** < 0.001**
**mRS at discharge (0–5)**^**c**^	**1 (0–1)**	**0 (0–0)**	** < 0.001**
Total length of the hospital stay (days)^b^	5.5 (3/1–18)	5.5 (2.8/1–13)	0.815
* Length of Stroke-Unit stay (days)*^b^	3.6 (3/0–14)	3.1 (2.3/0–10)	0.262

*RAO* Retinal artery occlusion, *CRAO* Central retinal artery occlusion, *BRAO* Branch retinal artery occlusion, *AF* Amaurosis fugax, *ASA* Acetylsalicylic acid, *DAPT* Dual antiplatelet therapy, *NIHSS* National Institutes of Health Stroke Scale, *mRS* Modified Rankin scale, *SD* Standard deviation, *IQR* interquartile range

^a^*n* (%)

^b^mean (SD/range)

^c^median (IQR)

### Subgroup analysis of patients with RAO who received IVT

A total of 18 patients (24%) with RAO received IVT within a median door to needle time of 52 min. Only 8 patients presented within the approved time-window for IVT. Regarding the analysis of ophthalmological follow-up (Table 4), visual acuity improved in 5 patients, worsened in 2 and remained unchanged in 9. In all 5 cases, however, the improvement was limited to an improvement of only a single point within the blindness category. Thus, no clinically relevant improvement was observed for any patient. Regarding timing (< 4.5 h vs. 4.5–12 h) of IVT in relation to ophthalmological outcome we did not detect significant differences.

**Table 4:** Subgroup analysis of patients with RAO who received IVT

Characteristics	RAO (*n* = 18) (CRAO ( *n* = 15)/BRAO ( *n* = 3))	
Age (years)^a^	68.50 (11.9/41–81)	
Female^a^	6 (33)	
Time from onset to admission^a^		
< *4,5 (hours)*	8 (44)	
* 4,5–12 (hours)*	10 (56)	
TOAST^a^		
* Large artery atherosclerosis*	5 (27.8)	
* Cardioembolism*	5 (27.8)	
* Small vessel disease*	2 (11.1)	
* Other determined cause*	3 (16.7)	
* Undetermined cause*	3 (16.7)	
Visual impairment (VI)^a^ on admission follow-up		
* Mild or no VI*	2 (11.1)	1 (6.3)
* Moderate VI*	0 (0)	2 (12.5)
* Severe VI*	1 (5.6)	1 (6.3)
* Blindness I*	4 (22.2)	4 (25)
* Blindness II*	9 (50)	8 (50)
* Blindness III*	2 (11.1)	0 (0)
Door-to-needle-time (minutes)^b^	52.2 (47.2/20–198)	

*RAO* Retinal artery occlusion, *CRAO* Central retinal artery occlusion, *BRAO* Branch retinal artery occlusion, *TOAST* Trial of Org 10,172 in Acute Stroke Treatment, *SD* Standard deviation, *IQR* interquartile range

^a^n (%)

^b^mean (SD/range)

## Discussion

This comparative study demonstrated that (i) RAO patients did not show more severe vascular comorbidities, while (ii) AF patients received vascular interventions significantly more often. Regarding (iii) pharmacological intensification there was no difference among both groups. (iv) Clinical neurological outcome was slightly worse in RAO patients. Finally, (v) IVT, irrespective of time window applied, did not lead to favorable ophthalmological outcomes in any of our patients. Three aspects emerge from the data.

First, both RAO and AF patients showed a similar vascular risk factor and comorbidity profile. This is an interesting finding in light of the discussion that both diseases may represent the ophthalmological TIA, or AIS, respectively [9]. In our study, patients with permanent and transient retinal artery occlusion were approximately the same age, had similar preexisting comorbidities, and had a similar cardiovascular risk profile. While the aspect of a certain vascular burden has been demonstrated previously for RAO patients [10, 25], the lacking imbalances of vascular burden compared to AF contrasts pertinent literature on cerebral ischemia. On the one hand, several reports verified that patients with TIA are younger and have fewer comorbidities than those with infarction [42]. On the other hand, however, risk for recurrent stroke is increased among both RAO and AF, which is in line to the situation in TIA and AIS [4, 12]. Thus, in light of a high chance of dealing with the same patient population in RAO and AF, both patient groups should be considered in the same way as TIA and AIS. This fact harbors clinical implications for daily clinical routine such as, that patients with RAO and AF should undergo the same dedicated diagnostic workup and potential management on a stroke unit. This however, at least for the group of RAO patients, is not established everywhere, as there are countries including Germany, in which RAO patients are often treated as out-patients. Stroke units not only allow rapid assessment but are also designed to cover early rehabilitation and monitoring for early recurrent strokes [24]. While patients with RAO and AF are less likely to benefit from the early rehabilitation capacity, a rapid assessment certainly seems appropriate. However, the structured and immediate work-up would not necessarily have to take place on a stroke unit. In principle, this could also be performed by peripheral units with a lower level of care and the appropriate expertise, in order to avoid unnecessary overloading of stroke units.

Of note, the different CRP levels on admission suit well into the ongoing debate of an underlying pro-inflammatory process as a contributing factor for stroke ictus [23]. In patients with AIS it has been established previously that elevated CRP levels on admission are related to poorer long term prognosis including all-cause mortality [45]. Future studies should focus on long-term clinical course of patients with RAO at a similar intensity as it is done currently in AIS patients [28, 29].

Second, regarding IVT in RAO, we did not observe any clinical benefit in any of the patients treated with IVT. This aspect is different to those patients who receive IVT in case of AIS, of whom a substantial proportion shows clinical improvement and thrombus resolution [43]. Studies suggest early thrombolysis for patients with RAO in order to increase the chance of revascularization of retinal vessels and thus the prevention of irreversible loss of vision [26], which is why this procedure is recommended by guidelines [13, 28, 29]. However, according to a Cochrane Review, there is a lack of high-level evidence to support this claim [18]. Regarding time-window for IVT, it was hypothesized to be possibly shorter in retinal ischemia compared to AIS [40]. While the postulated threshold of 4.5 h after symptom onset has extensive scientific support for patients with AIS, its relevance for patients with RAO is based on only a single animal study in 5 rhesus monkeys [21]. In AIS, there is a certain collateral flow in the penumbra of ischemic core of the cerebral infarction keeping neurons and Central-Nervous-System tissue salvageable for hours [8]. Although there seem to be similar retinal penumbra models and there might be significant inter-individual differences in retinal tolerance to ischemia [28, 29], the retinal ganglion cells that form the optic nerve appear to undergo apoptosis already after only 12–15 min [40]. Up to now, there have been no published results of the randomized IVT-trials in CRAO currently recruiting (NCT03197194, NCT04965038) why our understanding of time-windows in RAO will improve based on these data.

Third, in RAO there might be a substantial underreporting of transient vision losses given the benign course of disease [15]. This holds true for both AF-patients, but also to a significant amount in patients with CRAO/BRAO, notably in those who experience visual acuity improvement after minutes due to spontaneous recanalization or the presence of cilioretinal sparing [20]. These aspects require efforts to adopt heath and emergency care systems in various countries, including Germany. The lack of awareness of these diseases in the general population is reflected by a high number of patients in ophthalmological ED not being aware of the existence of these conditions [41]. There should be a significant escalation of public relations work in order to encourage people with transient vision loss to immediately seek emergency evaluation to verify or exclude RAO or AF. According to recent evidence, only a maximum of half of RAO patients regularly present to the ED within a time window for IVT [39]. Further, the typical patients´ contact with their ophthalmologists in this setting is not ideal, as it is associated with significant time delay for possible IVT.

Instead, emergency physicians, covering both neurological and ophthalmological expertise in the same ED, should handle both RAO as well as AF-patients. In addition, the respective professional neurological and ophthalmological societies should consent on a joint protocol for acute management of these patients including diagnostic workup by ophthalmologists paralleled by IVT-treatment by neurologists in order to save time and recruit more patients to the “golden hour”. These efforts should certainly result in an increased proportion of patients with correct diagnosis of RAO including an accelerated diagnostic and therapeutic workflow leading to more favorable clinical outcomes [9].

Our study has certain limitations, notably its monocentric and retrospective design that undermines the generalizability of our findings to other cohorts. Whilst it can be assumed that most patients with a persistent RAO receive a neurological examination at some point during the course of their disease, only an unknown proportion of patients with AF will probably ever be seen by an ophthalmologist or neurologist, due to the transient nature of their symptoms, similarly to TIA-patients [5]. Further, the etiology of AF should also be discussed. This study included patients whose symptoms were suspected to be due to underlying vascular disease, and therefore rapid vascular diagnostics seemed appropriate. Thus, the diagnosis of AF was based on the report of a sudden, painless, unilateral, and transient loss of vision with an attack duration of less than 24 h. However, as with TIA, there is a possibility that these symptoms may have been caused and mimicked by other diseases [35]. These considerations emphasize the need for better stratification of patients with transient monocular vision loss in the future. Another limitation is that only mRS scores were documented to assess patients' disability status. This measure is not ideal for stroke patients with isolated vision loss and was therefore not sensitive to differences during the course of hospitalization or between the two groups of patients. However, the mRS results indicated that both groups of patients generally had a low level of impairment in daily activities at both admission and discharge. This supports the assumption that patients with RAO or AF generally present with a low degree of neurological pre-injury and remain minimally impaired in the short term, i.e., within their inpatient stay.

## Conclusions

In conclusion, the present study verified similar vascular burden and risk factors in RAO- and AF-patients with implications for clinical and diagnostic workflows respectively. Further, IVT in RAO appears to be ineffective if not applied within very early time windows. These aspects should result in public relations work to increase awareness of RAO and AF as medical emergencies, and in a call for action to establish joint protocols of ophthalmologists and neurologists to improve clinical outcome.

## Data Availability

Requests for access to the data reported in this paper will be considered by the lead authors (NJD, HBH) depending on the terms of our regulatory approvals and institutional policies of individual study frame-works.

## Abbreviations

RAO: Retinal artery occlusion
CRAO: Central retinal artery occlusion
BRAO: Branch retinal artery occlusion
AIS: Acute ischemic stroke
IVT: Intravenous thrombolysis
AF: Amaurosis fugax
TIA: Transient ischemic attack
ED: Emergency department
A/PION: Anterior/posterior ischemic optic neuropathy
WHO: World Health Organization
NIHSS: National Institute of Health Stroke Scale
mRS: Modified Rankin Scale
TTE: Transthoracic echocardiography
TEE: Transesophageal echocardiography
PFO: Patent foramen ovale
EF: Ejection fraction
ICA: Internal carotid artery
CEA: Carotid endarterectomy
DAPT: Dual antiplatelet therapy
ASA: Acetylsalicylic acid
OAC: Oral anticoagulation
SD: Standard deviation
TOAST: Trial of Org 10172 in Acute Stroke Treatment
CRP: C-reactive protein
